# Experimental and computational evaluation of kolliphor RH 40 as a new fluorescence enhancer in development of a micellar-based spectrofluorimetric method for determination of lapatinib in tablets and urine

**DOI:** 10.1371/journal.pone.0239918

**Published:** 2020-12-03

**Authors:** Hany W. Darwish, Ahmed H. Bakheit, Nasser S. Al-shakliah, A. F. M. Motiur Rahman, Ibrahim A. Darwish

**Affiliations:** 1 Department of Pharmaceutical Chemistry, College of Pharmacy, King Saud University, Riyadh, Kingdom of Saudi Arabia; 2 Department of Analytical Chemistry Faculty of Pharmacy, Cairo University, Cairo, Egypt; 3 Department of Chemistry, Faculty of Science and Technology, Al-Neelain University, Khartoum, Sudan; 4 Department of Pharmaceutical Chemistry, College of Pharmacy, Aden University, Aden, Yemen; Bhagwan Mahvir College of Pharmacy, INDIA

## Abstract

This study describes, for the first time, the experimental and computational investigations for evaluation of kolliphor RH 40 as a fluorescence enhancer surfactant in development of a spectrofluorimetric method for determination of lapatinib (LAP), a tyrosine kinase-inhibitor drug approved for targeted therapy of breast cancer. The investigations involved the ability of kolliphor RH 40 to form micelles with LAP and its enhancing effect on the weak native fluorescence of LAP at 420 nm after its excitation at 292 nm. Different variables were experimentally investigated: types of organized media, diluting solvent, buffer type and its pH value. The optimum values of the most influencing variables on the interaction of kolliphor RH 40 with LAP were refined by the computational response surface methodology (RSM). Under the optimized conditions, it was found that kolliphor RH 40 forms micelles with LAP, and its fluorescence enhancing ability was higher than other surfactants tested by ~ 10-folds. This micellar-enhanced effect of kolliphor RH 40 was employed in the development of a new sensitive spectrofluorimetric method for the accurate determination of LAP. The method was validated according to the guidelines of the International Conference on Harmonization (ICH) for validation of analytical procedures. The relative fluorescence intensity (RFI) was in excellent linear relationship (correlation coefficient was 0.998) with the LAP concentrations in the range of 50–1000 ng/mL. The method limit of detection (LOD) was 27.31 ng/mL and its accuracy was ≥ 99.82%. The method was successfully applied to the determination of LAP in its pharmaceutical tablets, tablets dissolution testing and content uniformity. The method application was extended to the determination of LAP in urine samples with an accuracy of 99.82 ± 3.45%. The method is considered as an eco-friendly green approach and more efficient alternative method to the existing analytical methodologies for determination of LAP.

## Introduction

**Fig 1:**
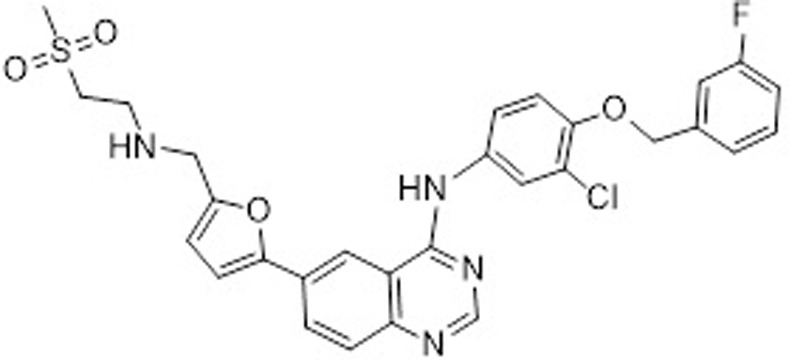
Chemical structure of lapatinib (LAP).

Lapatinib (LAP) is chemically named as N-{3-chloro-4-[(3-fluorobenzyl)oxy]phenyl}-6-[5-({[2-(methylsulfonyl)ethyl]amino}methyl)-2-furyl]-4-quinazolinamine; the chemical structure is given in Fig 1. The molecular formula of LAP is C_29_H_26_ClFN_4_O_4_S and its molecular mass is 581.057. LAP is a potent orally active drug against breast cancer and other solid tumors [1]. LAP, as ditosylate salt, has been first approved by the United States Food and Drug Administration (US-FDA) under the trade name of Tykerb^®^ (in USA) and Tyverb^®^ (mostly in Europe) as film-coated tablets by the pharmaceutical company GlaxoSmithKline (GSK). Subsequently, Tykerb^®^ has received an accelerated approval for the treatment of postmenopausal women with hormone receptor positive metastatic breast cancer that overexpresses the HER2 receptor and for whom hormonal therapy is indicated (in combination with letrozole) [2]. LAP has a dual tyrosine kinase inhibitor action which interrupts the HER2/neu and epidermal growth factor receptor (EGFR) pathways [3–7].

The oral bioavailability of LAP is moderate at best, interpatient variability is considerable and it is significantly affected by food intake as its systemic exposure increases by low/high fat diets intake before LAP administration [8, 9]. Gastric pH also influences its bioavailability as higher gastric pH causes reduction of LAP absorption; therefore, a concomitant use of proton-pump inhibitors can decrease its bioavailability [10]. Like many small molecule tyrosine kinase inhibitors, LAP is well tolerated; however it shows some side effects which mostly include diarrhea, fatigue), nausea and rashes [11].

LAP has been determined in its pharmaceutical tablets and/or biological fluids by liquid chromatography coupled with ultraviolet [12], diode array [13] and tandem mass spectrometric detectors [14–23]. Chromatographic techniques are selective and sensitive; however, they are laborious, tedious, time-consuming, and rely on expensive instruments which limit their routine use in quality control laboratories, particularly in developing countries. Spectrofluorimetric analysis is widely used in quality control laboratories because it is simple and sensitive technique; however, the technique has not yet described for determination of LAP. Therefore, the present study was devoted to development of new of a spectrofluorimetric method that can overcome the drawbacks of the existing liquid chromatographic methodologies. It was possible to derivatize LAP, via its secondary amino group, with different fluorescent reagents [24]. However, this approach usually requires high reaction temperature, long time, and occur in organic solvents which are costive and more importantly increase the incidence of exposure of the analysts to these toxic solvents [25–29]. For these reasons, investigating new alternative approach that give high fluorescence intensity and use minimum volumes of organic solvents, if any, was very important. Recently, spectrofluorimetric methods involving enhancement of fluorescence signal by micelle formation have been developed for the quantification of various drugs [30–33]. These micelle-enhanced spectrofluorimetric methods offered high sensitivities and considered as green eco-friendly approach as it occurs in safe aqueous media. For development of these methods, various surfactants could be used such as sodium dodecyl sulphate [30], tween [31] and cyclodextrin [32, 33].

Kolliphor RH 40 is a macrogolglycerol hydroxystearate derived from hydrogenated castor oil and ethylene oxide. It is mainly used as a solubilizer and emulsifier in the oral and topical liquid and semi-solid pharmaceutical dosage forms. It demonstrated exceptional chemical stability and scored highest on resistance to enzymatic digestion among various surfactants, highlighting its potential to enhance patient safety. It is also used in self- and microemulsifying drug delivery in combination with a co-solubilizer and/or co-solvent [34]. However, it has not been investigated as a micelle-forming surfactant and a fluorescence enhancer for LAP.

The present study describes the investigations and analytical studies to evaluate kolliphor RH 40 as a new micelle-forming surfactant and a fluorescence enhancer in the development of a simple and sensitive micelle-enhanced spectrofluorimetric method for determination of LAP in its dosage forms and urine.

## Experimental

### Instruments

Fluorescence spectra were recorded on a JASCO FP-8200 fluorescence spectrometer (JASCO Corporation, Japan) equipped with a 150 W Xenon lamp and 1 cm quartz cells. The slit widths for both the excitation and emission monochromators were set at 5.0 nm. All recorded spectra converted to ASCII format by Spectra Manager^TM^ software provided with the instrument. A Hanna pH-meter (Romania) was used for pH adjustments. Automatic dissolution tester, 8 cup system (Abbott Corporation, United States) was used for *in-vitro* drug release testing.

### Reagents and materials

Lapatinib (LAP) was purchased from LC Laboratories (Woburn, MA, USA); its purity was > 99.00%. Tykerb® tablets (GlaxoSmithKline, UK) labeled to contain 250 mg (as ditosylate monohydrate) per tablet were obtained from local Saudi market. Kolliphor RH 40 was purchased from BASF (Ludwigshafen, Germany); it was used as 2% (w/v) aqueous solution. Sodium dodecyl sulphate (SDS; Winlab, UK) was used as 2% (w/v) aqueous solution. Tween 20 and Tween 80 were purchased from Techno Pharmchem Haryana (India) and used as 2% (v/v) aqueous solutions. Triton was purchased from Loba Chemie (India) and used as 2% (v/v) aqueous solution. Β-Cyclodextrin (β-CYCD; Merck, Germany) was used as 2% (w/v) aqueous solution. Methanol and ethanol were obtained from Prolabo (France). Acetonitrile was obtained from Sigma-Aldrich (St. Louis, CA, USA). Boric acid, citric acid, sodium hydroxide, phosphoric acid, potassium chloride, potassium dihydrogen phosphate and disodium hydrogen phosphate were of analytical grade. Water was used after purification by Milli-Q plus instrument (USA). Phosphate buffer (0.1 M, pH 2–12), citrate buffer (0.1 M, pH 3–7), and borate buffer (0.1 M, pH 8–10) solutions were freshly prepared before use. Human urine was kindly supplied by King Khaled University Hospital (King Saud University, Riyadh, KSA) following informed verbal consent. Urine samples were collected, centrifuged, and stored at -70°C until analysis.

### Preparation of standard solutions

Stock LAP standard solution (10 mg mL^−1^) was prepared by dissolving 250 mg of LAP standard material in 25 mL of acetonitrile. Working LAP solution (1 μg mL^−1^) was prepared from the stock solution by dilution in the same solvent.

### Optimization of experimental conditions

Box–Behnken 3^3^experimental design with Design Expert software (version 7.1.1; Stat-Ease, Inc., USA) was used in establishing the optimum values for the most influencing factors on the interaction of kolliphor RH 40 with LAP. These factors were pH, volume of buffer solution and concentration of the kolliphor RH 40 surfactant. Other experimental conditions (measuring wavelengths, and diluting solvent) were kept constant at their optimum values determined by the practical experiments. Optimization procedure involved statistical design for the response related-combinations, adjusting the practical results with the computational responses, and define the suitability of the model. The variable's levels were selected depending on their least and highest influence on the relative fluorescence intensity (RFI) and similarly each level was experienced at lower and upper value.

### General analytical procedure

Calibration samples in the range of 50–1000 ng/mL were prepared from standard solution of LAP. In a 5-mL volumetric flask, 0.5 mL of the LAP calibration sample was transferred. To this solution, 2 mL of kolliphor RH 40 (2%, w/v) and 2 mL of phosphate buffer (PB) solution of pH 12 were added, and the volume was completed with water. The contents were mixed well and RFI was measured at 420 nm for emission after excitation at 292 nm. The measured RFI values were plotted as a function of LAP concentrations. Regression analysis of the data was conducted and the regression equation was computed, from which the unknown concentrations of LAP samples were derived.

### Determination of LAP in tablet samples

Twenty Tykerb^®^ tablets (Lot no. R663914) were weighed, crashed to fine powder. From the tablet powder, an accurately weighed quantity (250 mg) was transferred to 25-mL volumetric flask and dissolved in ~ 20 mL of acetonitrile. The solution was sonicated for 30 min to ensure the complete dissolution of LAP, and the volume was completed to the mark with the same solvent. This tablet solution (claimed to be 10 mg/mL) was filtered with Chromafil^®^Xtra 0.2 μm filter papers and diluted with acetonitrile to give concentrations in the range of 50–1000 ng/mL. These diluted samples were subjected to the analysis by the proposed method as described under “General analytical procedure”.

### Procedure for content uniformity testing for Tykerb® tablets

Ten Tykerb^®^ tablets were analyzed for their contents of LAP by applying the procedures described under “Determination of LAP in tablet samples”. Guidelines of the United States Pharmacopeia (USP) [35] (chapter 905: Uniformity of Dosage Units) were followed for the uniformity testing of the Tykerb^®^ tablets contents. According to official USP guidelines, the content uniformity could be performed by computing the acceptance value (AV) for tablets. Average was measured by applying the equation: AV = |H–$\overset{-}{X}$| + KS, where; H is a reference value, $\overset{-}{X}$ is the individual content average, K is the acceptability constant, and S is the standard deviation of samples. If average for ten tablets ≤ maximum allowed acceptance value, thus the content uniformity is accepted (as in the present study).

### Procedure for in-vitro drug release test (dissolution test) for LAP

*In-vitro* release of Tykerb^®^ tablets was conducted according to the FDA procedure of dissolution [36]. A volume of 900 mL of dissolution solution (2% of polysorbate in 0.1 N HCl) was kept at ambient temperature, stirred at a speed of 55 rpm for 60 min., and used for the dissolution of the Tykerb^®^ tablets. Aliquots (5 mL) of Tykerb^®^ tablet solution were withdrawn at time intervals (10, 15, 30, 45 and 60 min) and filtered using 0.45 μm syringe filter. The volume of dissolution medium was kept constant by replacement of the withdrawn sample volume by equal volume of the dissolution medium. The samples were subjected to the analysis for their LAP contents by the proposed method as described under “General Analytical Procedure.

### Determination of LAP in human urine samples

Human urine samples (2 mL) were spiked with 50 *μ*L of LAP standard solution containing varying concentrations of LAP, and the contents were mixed by vorex for 30 sec. To this mixed solution, 1 mL of NaOH 100 mM/glycine buffer (pH 11) was added and the contents were mixed for 10 sec. These samples were subjected to liquid-liquid extraction with diethyl ether (5 mL) followed by centrifugation for 15 min at 10,000 rpm for phases separation. The upper organic layer (3 mL) was collected into a glass vial, dried over gentle nitrogen stream, and the obtained residue was reconstituted in acetonitrile to give final LAP concentrations of 375, 750, 1500, and 3000 ng/mL. These samples were analyzed for their LAP contents as described under “General Analytical Procedure. Blank urine samples were subjected to the same procedure whoever LAP spiking step was omitted. The study has been approved by the research ethics committee at King Saud University.

## Results and discussion

### Strategy of the study and spectral characteristics

**Fig 2:**
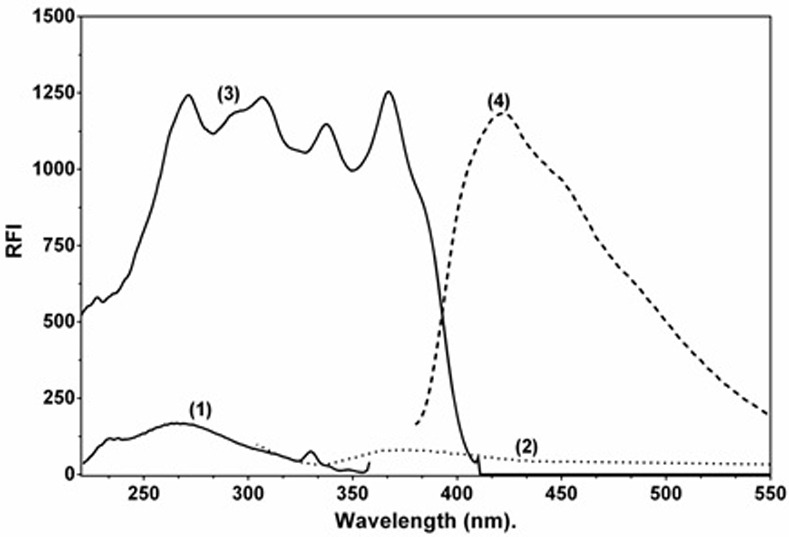
Fluorescence spectra of LAP (1000 ng/mL) and its interaction mixture with kolliphor RH 40 (2%, w/v). Spectra (1) and (2) are the excitation and emission (respectively) of LAP aqueous solution in absence of kolliphor RH 40. Spectra (3) and (4) are the excitation and emission (respectively) of LAP aqueous solution containing kolliphor RH 40.

LAP was selected for this study because of its therapeutic importance and the need for a simple and sensitive spectrofluorimetric method for its determination in tablets and biological samples. LAP exhibits very weak native fluorescence at emission and excitation wavelengths of 292 and 420 nm, respectively (Fig 2), thus its direct determination was not practically possible. Therefore, enhancement of its native fluorescence was necessary to increase its detectability and ultimately the sensitivity of its spectrofluorimetric determination. Development of micelle-based spectrofluorimetric method for LAP was considered because it combines the advantages of simplicity of the procedures, providing high sensitivity, and increasing the greenness of the approach. Kolliphor RH 40 was selected for the present study because it has not been investigated yet as a micelle-forming surfactant and fluorescence enhancer for LAP. Our preliminary experiments showed that addition of kolliphor RH 40 to LAP greatly enhanced the fluorescence of LAP at its emission and excitation wavelengths which were 292 and 420 nm, respectively (Fig 2). This increase in fluorescence intensity of LAP in the cited surfactant may be attributed to the protection of the lowermost excited singlet state from non-radiative procedures in the formed micelle [37, 40].

The use of response surface methodology (RSM) [38, 39] in more powerful technique than the conventional practical experiments. It provides a large amount of information and reduces the number of practical experiments. For these reasons, RSM was considered and used to find the optimum conditions for the interaction of kolliphor RH 40 with LAP in the present study. The present study was devoted to investigate the optimum conditions for use of kolliphor RH 40 in the development of a micellar-enhanced spectrofluorimetric method for determination of LAP.

### Practical optimization of the experimental conditions

The factors affecting the fluorescence intensity (concentration of kolliphor RH 40, type of organized media, buffer pH and volume, and the diluting solvent) were investigated by altering each variable by turn while keeping the others constant. All experiments were conducted on a working solution of LAP at a concentration of 1000 ng/mL, and all the fluorescence emission measurements in this study were carried out at 420 nm after excitation at 292 nm.

#### Effect of kolliphor RH 40 concentration

**Fig 3:**
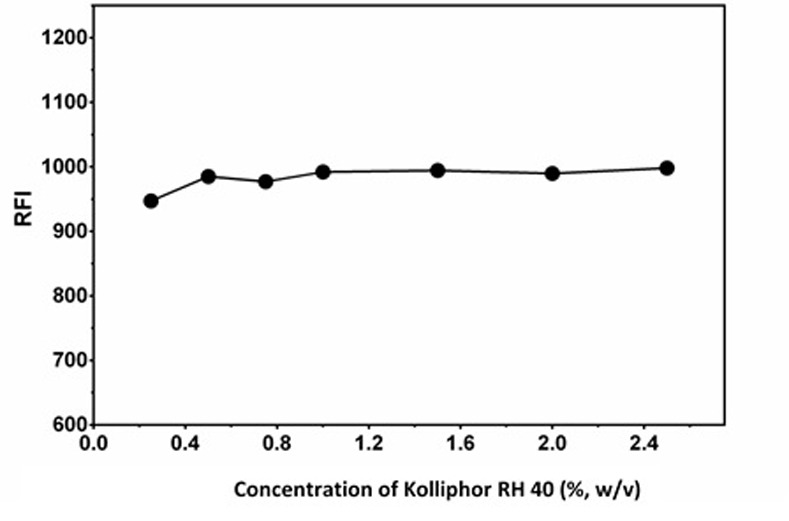
Effect of concentration of kolliphor RH 40 on the RFI values of its interaction mixture with LAP (1000 ng/mL).

The effect of concentration of kolliphor RH 40 was investigated by adding 2 mL of varying concentrations of kolliphor RH 40 in the range of 0.2–2.5% (w/v) to the LAP solution and the relative fluorescence intensity (RFI) was measured for each concentration. The results revealed that a concentration of 0.5% was adequate to give the highest RFI, beyond which the RFI values did not increase with the increase in kolliphor RH 40 concentrations (Fig 3). However, for readings with better precision and RSM results (discussed below), 2% (w/v) was used in the subsequent experiments.

#### Effect of type of organized media

**Fig 4:**
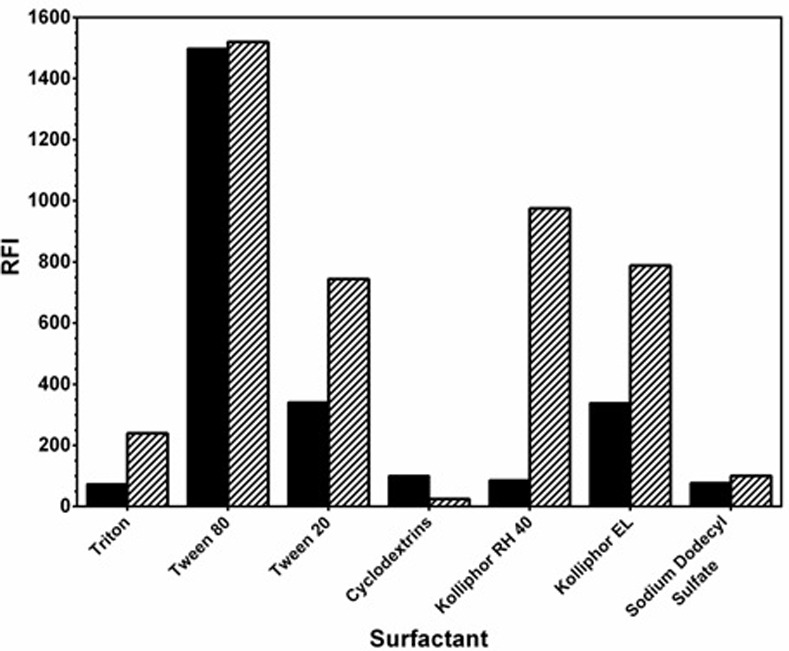
Effect of type of surfactant (1mL 1% w/v solution of each) on the RFI values of their interaction mixture with LAP (1000 ng/mL). Solid black bars: kolliphor RH 40, stripped bars: mixture of LAP and kolliphor RH 40.

Various organized media (surfactants solutions) were investigated and their effects on the RFI values were compared with those of kolliphor RH 40; 2 mL of equal concentrations (2%, w/v) was used for each. Among all the tested surfactants, kolliphor RH 40 gave the highest RFI values when mixed with LAP compared with those in absence of LAP (Fig 4). It is wise to mention that all surfactants gave fluorescence except cyclodextrins which may be due to formation of inclusion complexes, rather than micelles.

#### Effect of pH

**Fig 5:**
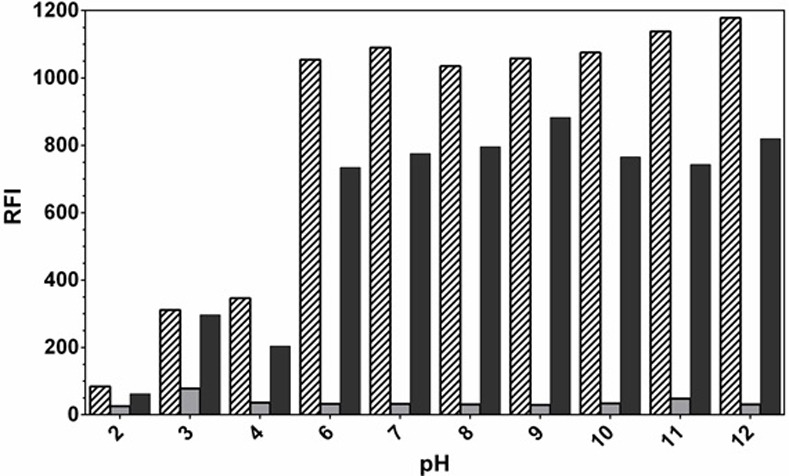
Effect of pH on the RFI values of the interaction mixture of kolliphor RH 40 (2%, w/v) with LAP (1000 ng/mL). Solid black bars: LAP, solid grey bars: kolliphor RH 40, stripped bars: mixture of LAP and kolliphor RH 40.

**Fig 6:**
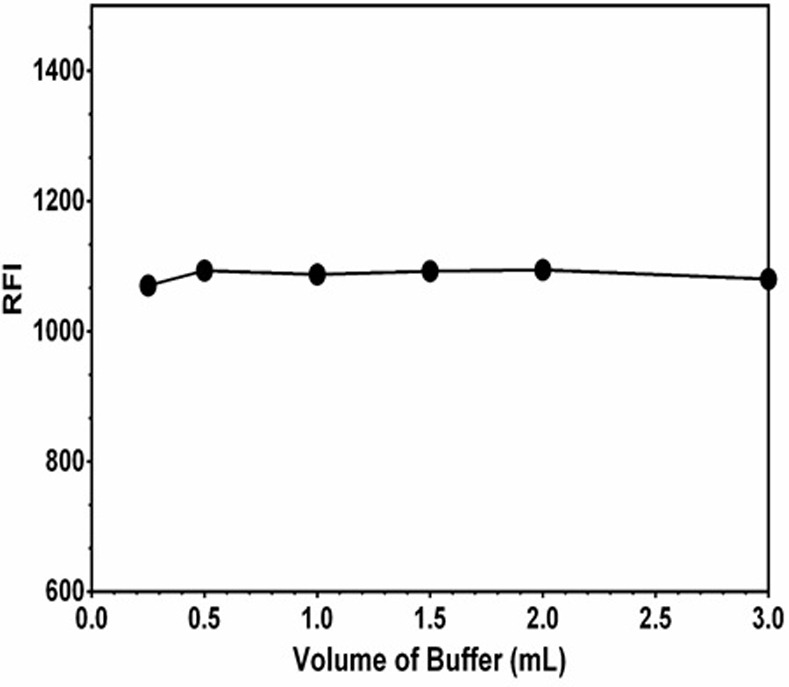
Effect of volume of phosphate buffer solution (pH 12) on the RFI values of the interaction mixture of LAP (1000 ng/mL) with kolliphor (2%, w/v).

For investigating the effect of pH, different buffers of varying pH values (2–12) were tested. The results indicated that the RFI values were pH dependent (Fig 5). In high acidic pH values (2–4), the RFI values were significantly lower than those obtained in neutral-alkaline pH values; the highest RFI values were obtained at pH 12. The volume of buffer solution did not affect the RFI values (Fig 6). In order to investigate the effect of buffer components on the RFI values, different buffers (phosphate, citrate, and borate) of similar pH values were tested. It was found that the RFI values were not affected by the type of buffer indicating that buffer components have no effect rather than the pH itself.

**Fig 7:**
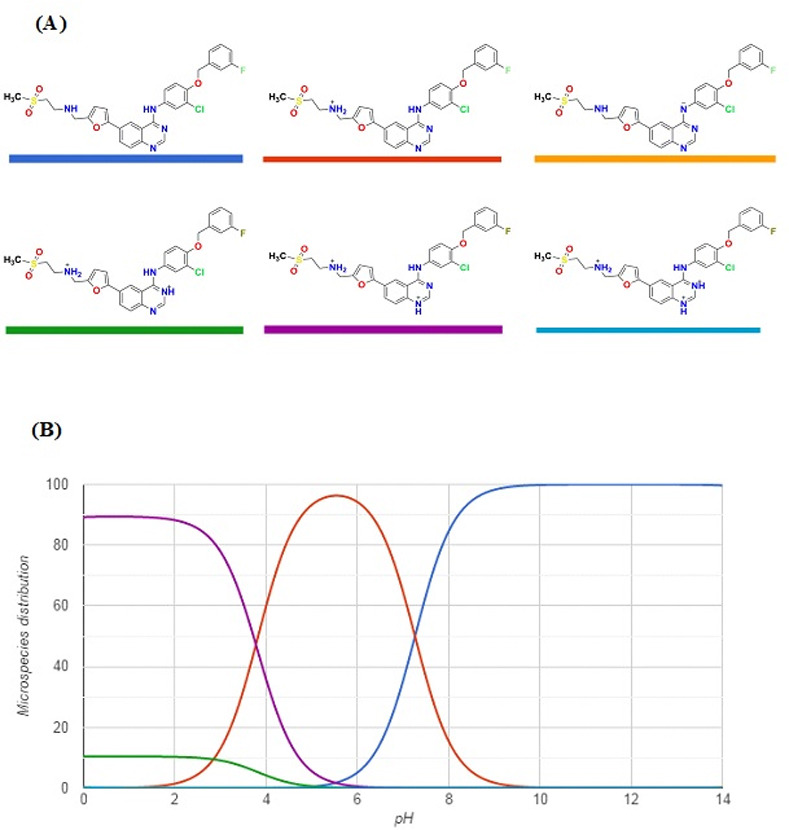
Theoretical protonation states of LAP as a function of the pH. Panel (A): the chemical structures of the different protonated states of LAP. Panel (B): Microspecies distribution of LAP according to the pH value. The colors of the curves match with those of the chemical structures.

In order to figure out the effect of pH value on the interactions of LAP with kolliphor RH 40, the physicochemical properties of LAP were calculated by Chemicalize [40]. It was found that LAP molecule can exist as six different protonated states (Fig 7A). The only predominant state of LAP is the non-ionic one which exists at pH 12 (Fig 7B). This explains the strong interaction between LAP and kolliphor RH 40 at pH 12.

#### Effect of diluting solvent

**Fig 8:**
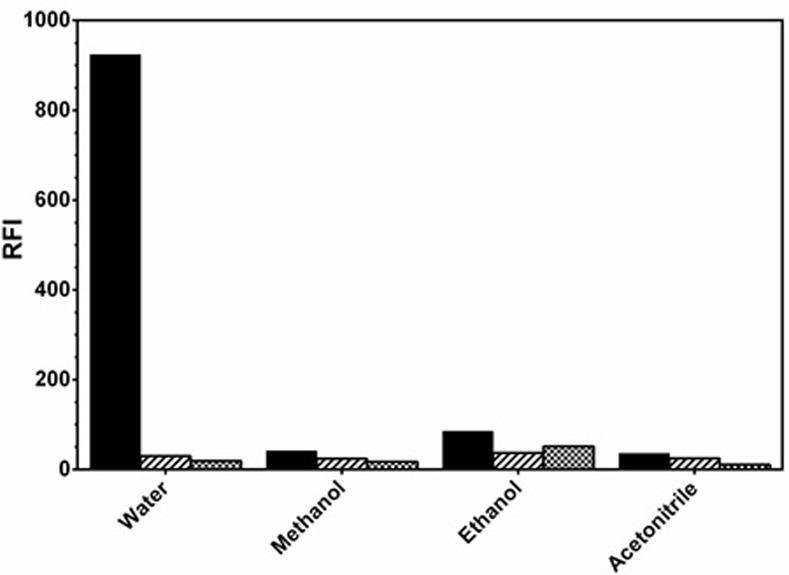
Effect of diluting solvent on the RFI values of the interaction mixture of LAP (1000 ng/mL) with kolliphor RH 40 (2%, w/v). Dotted bars: kolliphor RH 40, stripped bars: LAP, solid black bars: mixture of LAP and kolliphor RH 40.

The buffered interaction mixture of LAP and kolliphor RH 40 was diluted with various solvent (water, ethanol, methanol and acetonitrile), and RFI values were measured in each case. The highest RFI readings were obtained when water was used for dilution (Fig 8). Dilution with water gave an advantage to the method as it is cheap, and more importantly eco-friendly. Additionally, the observed remarkable decrease in the RFI on utilizing methanol, acetonitrile or ethanol may be due to the denaturating influence of these organic solvents on the micelle formation and may, at high concentration, breakdown the surfactant aggregate.

### Response surface methodology for optimization of conditions

**Fig 9:**
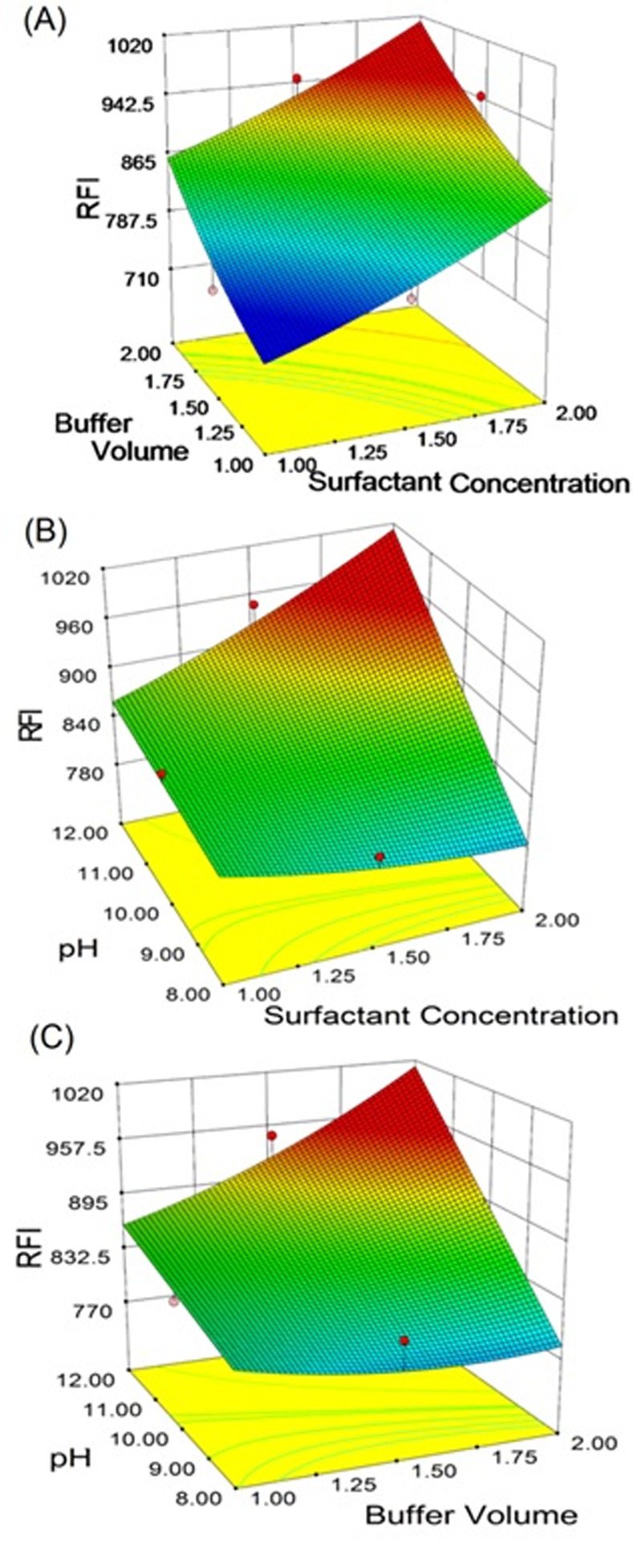
Response surface graphs showing the influence of kolliphor RH 40 surfactant concentration, volume of buffer and buffer pH on RFI values of the interaction mixture with LAP. Panel (A): effect of surfactant concentration and buffer volume on RFI of LAP while keeping pH constant. Panel (B): effect of surfactant concentration and buffer pH on RFI of LAP, while keeping buffer volume constant. Panel (C): effect of buffer volume and pH on RFI of LAP, while keeping surfactant concentration constant.

As evident from the above mentioned practical optimization of the conditions affecting the interaction of kolliphor RH 40 with LAP were the pH and volume of buffer solution, concentration of the kolliphor RH 40, RSM were adopted for refining the optimum values of these conditions. Analysis of the collected data was performed by Design Expert software and nonlinear quadratic models for data fitting for LAP quantification. Fig 9A–9C illustrate the response surface graphs for best fitted model. Point prediction tool (embedded in the software) was utilized for RFI calculation. The results revealed that the optimum values for the three studied parameters were 12 for pH value, 2 mL for buffer volume and 2% (w/v) for concentration of kolliphor RH 40 surfactant. The effect of surfactant concentration and buffer on the response at constant pH is shown in Fig 9A. It is obvious that the RFI increased with increasing surfactant concentration. The effect of surfactant concentration and pH on LAP response at constant buffer volume is depicted in Fig 9B showing that the increase in surfactant concentration at higher pH value augmented LAP response. Finally, Fig 9C showed the effect of buffer volume and pH on LAP response at constant surfactant concentration (2%, w/v) and it was clear that as pH and buffer volume increased, the RFI of LAP increased. These optimized values were used in the quantitative determinations of LAP in its different samples.

### Validation of the method

#### Linear range and sensitivity

Calibration curve was constructed by plotting the RFI values (on *y-*axis) as a function of their corresponding LAP concentrations (on *x-*axis, in ng/mL). Regression analysis for the data was conducted [41] and it was found that the curve was linear in the range of 50–1000 ng/mL; as evident from the good correlation coefficient value of 0.998, and the other parameters of the regression analysis (Table 1).

**Table 1:** Analytical data of the micellar-enhanced spectrofluorimetric method for determination of LAP.

Parameter	Value
Wavelength; λ_ex/_λ_em_ (nm)	292/420
Linearity range (ng/mL)	50–1000
Intercept *(a)*	-13.48
Slope *(b)*	0.9046
Correlation coefficient (*r*)	0.998
Standard deviation of residuals (S_y/x_)	20.870
Standard deviation of intercept (S_a_)	± 7.486
Standard deviation of slope (S_b_)	± 0.013
Relative standard deviation (%)	1.68
Error (%)	1.11
Limit of detection (ng/mL)	27.31
Limit of quantitation (ng/mL)	82.75

The limit of detection (LOD) and limit of quantitation (LOQ) were calculated using reported ICH Q2 (R1) recommendations [42] using the following formula:

$$\text{LOD}=3.3\times \frac{Sa}{b}\;and\;LOQ=10\times \frac{Sa}{b}$$

Where, *Sa* of the intercept of regression line of calibration curve; *b* = the slope of regression line of the calibration curve (Table 1). LOD and LOQ values were found to be 27.31 and 82.75 ng/mL, respectively (Table 1).

#### Accuracy and precision

For the determination of the accuracy of this spectrofluorimetric method, different samples of bulk powder and Tykerb^®^ tablets claimed to contain varying concentrations of LAP were analyzed. As shown in Table 2, the mean recovery values were values were 99.93 ± 2.36 and 102.41 ± 2.05%, respectively indicating the accuracy of the spectrofluorimetric method for determination of LAP in its bulk form and tablets. Similarly, urine samples were spiked with varying concentrations of LAP and subjected to the analysis by the proposed method. The mean recovery value was 99.82 ± 3.45% indicating the accuracy of the method for determination of LAP in its urine samples (Table 2).

**Table 2:** Determination of LAP in bulk powder, Tykerb^®^ tablets and urine by the proposed method.

Bulk powder			Tykerb tablets			Urine samples		
Taken conc. (ng/mL)	Found conc. (ng/mL)	Recovery (%)	Taken conc. (ng/mL)	Found conc. (ng/mL)	Recovery (%)	Added conc. (ng/mL)	Found conc. (ng/mL)	Recovery (%)
100	102.58	102.58	100	104.05	104.05	375	379.81	101.28
400	291.22	97.07	300	418.44	104.61	750	748.92	99.85
600	597.88	99.65	600	611.93	101.98	1500	1546.70	103.11
800	816.19	102.02	800	816.11	102.01	3000	2851.14	95.03
1000	983.22	98.32	1000	994.00	99.40			
	Mean	99.93			102.41		Mean	99.82
	± SD	2.36			2.05		± SD	3.45

Various concentrations (100–800 ng/mL) of LAP were analyzed, in triplicate, to evaluate the intra- and inter-assay precision. The relative standard deviation (RSD) values were 2.15–3.44 and 0.48–2.74% for the intra- and inter-assay precisions, respectively (Table 3). These low RSD values confirmed the high precision of the method. In addition, the accuracy of the spectrofluorimetric method adopted was verified by comparing the results of the determination of LAP with the results of the reported LC method [13] in pure form. Calculated F and t- values were lower than the tabulated values (Table 4), which suggested that there were no significant differences between the precision and accuracy of the adopted and reported methods.

**Table 3:** Accuracy and precision of the proposed micellar-enhanced spectrofluorimetric method for determination of LAP.

Taken conc. (ng/mL)	Recovery (%)	RSD (%)	Error (%)
Intra-day			
100	102.70 ± 2.36	2.30	2.70
200	103.20 ± 2.53	3.42	3.20
600	101.57 ± 2.18	2.15	1.57
800	102.16 ± 2.50	2.44	2.16
Inter-day			
100	104.05 ± 2.86	2.74	4.05
400	104.61 ± 0.65	0.62	4.61
600	101.98 ± 2.38	2.34	1.98
800	102.01 ± 3.72	0.48	2.01

**Table 4:** Statistical comparison of the results obtained by the proposed method and the reference method [13] for the determination of LAP in bulk powder.

Parameters	Proposed method	Reported method [13]
Mean	99.93	102.11
SD	2.360	1.671
RSD	2.362	1.636
Variance	5.570	2.791
n	5	5
Student’s t-test*(2.306)	1.690	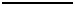
F- test*(6.388)	1.992	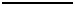

* Figures in parenthesis are the corresponding tabulated values at *p* = 0.05.

#### Robustness and specificity

The experimental conditions were deliberately changed and the effects of the changes on the analytical results obtained in each case are summarized in Table 5. As evident from the results, little changes that presumably occur throughout the experimental trail would do not have any vital effect on analytical results of LAP analysis as the recovery values were in the range of 97.77–104.97% (± 0.07–3.00). These good recovery values with low RSD indicated the robustness of the method.

**Table 5:** Robustness of the proposed micellar-enhanced spectrofluorimetric method for determination of LAP.

Experimental Parameter	Recovery (% ± SD) ^a^
No variation ^b^	99.4 ± 0.48
Kolliphor RH 40 volume (μL)	
950	99.29 ± 1.47
1050	99.13 ± 3.00
pH variation	
11.7	98.87 ± 1.37
12.3	104.97 ± 2.50
Buffer volume (μL)	
950	97.77 ± 2.69
1050	100.97 ± 0.07

^a^ Mean of three determinations.

^b^ General analytical procedure was used.

The specificity of the proposed spectrofluorimetric method was assessed by studying the potential interferences of the inactive ingredients (in tablets) or endogenous compounds (in urine samples). Good recovery values were obtained in both cases; these values were 99.40 –104.61 and 95.03–103.11% for tablets and urine samples, respectively (Table 2). These good recovery values indicated the specificity of the method for determination of LAP in both samples without interferences from tablet excipients or urine matrix.

### Applications of the proposed method

#### Analysis of Tykerb® tablets and content uniformity testing

Tykerb^®^ tablets were subjected to the analysis by the proposed spectrofluorimetric method. The mean label percentage value was found to be 102.11 ± 2.62% (Table 6). For the content uniformity test of Tykerb^®^ tablets, USP guidelines [35] were followed. While testing content uniformity of Tykerb^®^ tablet using this method, it was found that the method is rapid and simple. From the data inserted in Table 5, acceptance value was 5.67 is less than the maximum allowed average value of 15, Thus, the results revealed excellent drug uniformity.

**Table 6:** Content uniformity testing of Tykerb^®^ tablets using the micellar-enhanced spectrofluorimetric method.

Tablet no.	Label claim (%)
1	99.65 ± 1.91
2	97.82 ± 3.72
3	102.61 ± 0.52
4	104.94 ±1.75
5	106.76 ± 3.02
6	103.70 ± 2.73
7	100.01 ± 1.42
8	97.13 ± 2.83
9	104.11 ± 3.90
10	104.40 ± 3.02
Mean	102.11
SD	2.62
RSD (%)	2.57
Acceptance value	5.67
Max. allowed value	15

#### In-vitro drug release of Tykerb® tablets

**Fig 10:**
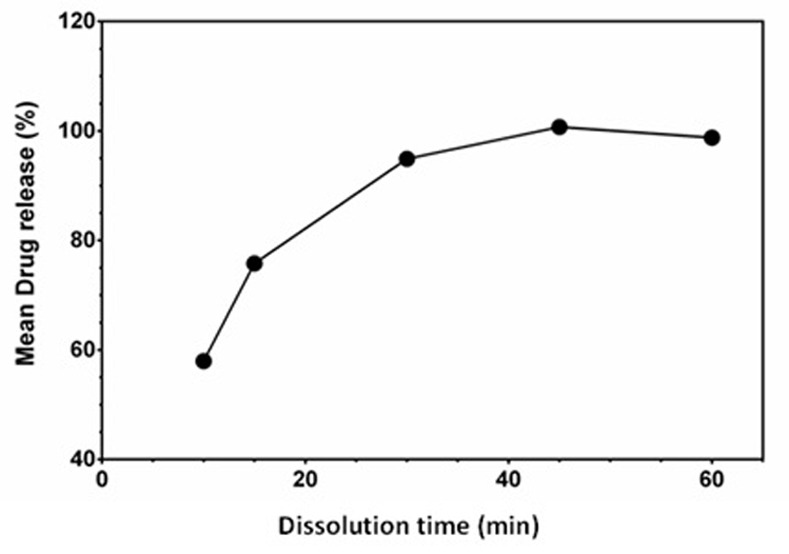
Effect of time on the dissolution of Lap tablets.

*In-vitro* drug release test (dissolution test) was performed on Tykerb^®^ tablet (250 mg). RFI of LAP at 420 nm in the regression linear equation was used to determinate the concentration of the released LAP. The amount of drug released (expressed as percentages) was monitored with the dissolution time (Table 7). It was found that more than 90% of LAP was released within 30 min, which is acceptable according to the USP guidelines (Fig 10).

**Table 7:** Data for the *in-vitro* dissolution test for Tykerb^®^ tablets.

Time (min.)	Mean drug release (%)
10	57.96
15	75.78
30	94.90
45	100.70
60	98.76

#### Analysis of LAP in urine

The amount of LAP excreted in urine as unchanged was estimated as 20–25 mg/day. Accordingly, these concentrations of LAP were spiked in human urine samples and the treated extracted samples were diluted to the get the concentrations in the linear range of the method (375–3000 ng/mL). The obtained average recovery of LAP was 99.82 ± 3.45% (Table 2).

## Conclusions

Experimental and computational investigations confirmed the validity of kolliphor RH 40 as a new powerful micelle-forming and fluorescence enhancer surfactant for LAP. Its fluorescence enhancing ability was higher than other surfactants tested by ~ 10-folds. Kolliphor RH 40 was successfully employed in the development of a micellar-enhanced spectrofluorimetric method for determination of LAP. The method is sensitive as it was able to quantitate as low LAP concentrations as 82.75 ng/mL and its accuracy was ≥ 99.82%. The method was successfully applied to the determination of LAP in its tablets and in human urine samples without interferences from the tablet excipients or from the endogenous urine matrix. The proposed method is characterized by rapidness and simplicity when compared with the reported chromatographic methods for LAP. Additionally, the method is inexpensive as depends on measuring the enhanced native fluorescence of LAP and thus no need for using costly derivatizing reagents and/or drastic derivatization conditions. Furthermore, optimization of the key factors affecting RFI of LAP, RSM was adopted using Box–Behnken design. The method is considered as an eco-friendly green approach and more efficient alternative method to the existing analytical methodologies for determination of LAP.

## References

[1] Higa GM, Abraham J. Lapatinib in the treatment of breast cancer. Expert Rev Anticancer Ther. 2007;7(9):1183–92. 10.1586/14737140.7.9.1183 17892419

[2] Drugs.com, FDA Approves tykerb for first-line combination treatment of metastatic breast cancer, https://www.drugs.com/newdrugs/gsk-s-tykerb-receives-accelerated-approval-first-line-combination-metastatic-breast-cancer-1979.html, (2010) (accessed 3 February 2019).

[3] Tevaarwerk AJ, Kolesar JM. Lapatinib: A small-molecule inhibitor of epidermal growth factor receptor and human epidermal growth factor receptor—2 tyrosine kinases used in the treatment of breast cancer. Clin. Ther. 2009;31:2332–48. 10.1016/j.clinthera.2009.11.029 20110044

[4] Wan X, Zheng X, Pang X, Zhang Z, Zhang Q. Incorporation of lapatinib into human serum albumin nanoparticles with enhanced anti-tumor effects in HER2-positive breast cancer. Colloids Surf B Biointerfaces. 2015;136:817–27. 10.1016/j.colsurfb.2015.10.018 26539808

[5] Arteaga CL, Sliwkowski MX, Osborne CK, Perez EA, Puglisi F, Gianni L. Treatment of HER2-positive breast cancer: current status and future perspectives. Nat Rev Clin Oncol. 2012;9(1):16. 10.1038/nrclinonc.2011.177 22124364

[6] Tsang RY, Sadeghi S, Finn RS. Lapatinib, a dual-targeted small molecule inhibitor of EGFR and HER2, in HER2-amplified breast cancer: from bench to bedside. Clin. Med. Insights Ther. 2011;3:CMT. S3783.

[7] Wetterskog D, Shiu K, Chong I, Meijer T, Mackay A, Lambros M, et al Identification of novel determinants of resistance to lapatinib in ERBB2-amplified cancers. Oncogene. 2014;33(8):966 10.1038/onc.2013.41 23474757

[8] Burris HA, Taylor CW, Jones SF, Koch KM, Versola MJ, Arya N, et al A phase I and pharmacokinetic study of oral lapatinib administered once or twice daily in patients with solid malignancies. Clin Cancer Res. 2009;15(21):6702–8. 10.1158/1078-0432.CCR-09-0369 19825948 PMC3232441

[9] Devriese LA, Koch KM, Mergui-Roelvink M, Matthys GM, Ma WW, Robidoux A, et al Effects of low-fat and high-fat meals on steady-state pharmacokinetics of lapatinib in patients with advanced solid tumours. Invest New Drugs. 2014;32(3):481–8. 10.1007/s10637-013-0055-4 24346280

[10] Koch KM, Im YH, Kim SB, Urruticoechea Ribate A, Stephenson J, Botbyl J, et al Effects of esomeprazole on the pharmacokinetics of lapatinib in breast cancer patients. Clin Pharmacol Drug Dev. 2013;2(4):336–41. 10.1002/cpdd.45 27121938

[11] Burris HA III, Hurwitz HI, Dees EC, Dowlati A, Blackwell KL, O'Neil B, et al Phase I safety, pharmacokinetics, and clinical activity study of lapatinib (GW572016), a reversible dual inhibitor of epidermal growth factor receptor tyrosine kinases, in heavily pretreated patients with metastatic carcinomas. J Clin Oncol. 2005;23(23):5305–13. 10.1200/JCO.2005.16.584 15955900

[12] Escudero-Ortiz V, Pérez-Ruixo JJ, Valenzuela B. Development and validation of a high-performance liquid chromatography ultraviolet method for lapatinib quantification in human plasma. Ther Drug Monit. 2013;35(6):796–802. 10.1097/FTD.0b013e3182959080 23942544

[13] Saadat E, Dehghan Kelishady P, Ravar F, Kobarfard F, Dorkoosh FA. Development and validation of rapid stability-indicating RP-HPLC-DAD method for the quantification of lapatinib and mass spectrometry analysis of degraded products. J Chromatogr Sci. 2014;53(6):932–9. 10.1093/chromsci/bmu150 25491314

[14] Haouala A, Zanolari B, Rochat B, Montemurro M, Zaman K, Duchosal M, et al Therapeutic drug monitoring of the new targeted anticancer agents imatinib, nilotinib, dasatinib, sunitinib, sorafenib and lapatinib by LC tandem mass spectrometry. J Chromatogr B Analyt Technol Biomed Life Sci. 2009;877(22):1982–96. 10.1016/j.jchromb.2009.04.045 19505856

[15] Götze L, Hegele A, Metzelder SK, Renz H, Nockher WA. Development and clinical application of a LC-MS/MS method for simultaneous determination of various tyrosine kinase inhibitors in human plasma. Clin Chim Acta. 2012;413(1–2):143–9. 10.1016/j.cca.2011.09.012 21945732

[16] Lankheet NA, Hillebrand MJ, Rosing H, Schellens JH, Beijnen JH, Huitema AD. Method development and validation for the quantification of dasatinib, erlotinib, gefitinib, imatinib, lapatinib, nilotinib, sorafenib and sunitinib in human plasma by liquid chromatography coupled with tandem mass spectrometry. Biomed Chromatogr. 2013;27(4):466–76. 10.1002/bmc.2814 22987603

[17] Couchman L, Birch M, Ireland R, Corrigan A, Wickramasinghe S, Josephs D, et al An automated method for the measurement of a range of tyrosine kinase inhibitors in human plasma or serum using turbulent flow liquid chromatography–tandem mass spectrometry. Anal bioanal chem. 2012;403(6):1685–95. 10.1007/s00216-012-5970-2 22526649

[18] Bouchet S, Chauzit E, Ducint D, Castaing N, Canal-Raffin M, Moore N, et al Simultaneous determination of nine tyrosine kinase inhibitors by 96-well solid-phase extraction and ultra performance LC/MS-MS. Clin Chim Acta. 2011;412(11–12):1060–7. 10.1016/j.cca.2011.02.023 21345336

[19] van Dyk M, Miners JO, Kichenadasse G, McKinnon RA, Rowland A. A novel approach for the simultaneous quantification of 18 small molecule kinase inhibitors in human plasma: a platform for optimised KI dosing. J Chromatogr B Analyt Technol Biomed Life Sci. 2016;1033:17–26. 10.1016/j.jchromb.2016.07.046 27521531

[20] Andriamanana I, Gana I, Duretz B, Hulin A. Simultaneous analysis of anticancer agents bortezomib, imatinib, nilotinib, dasatinib, erlotinib, lapatinib, sorafenib, sunitinib and vandetanib in human plasma using LC/MS/MS. J Chromatogr B Analyt Technol Biomed Life Sci. 2013;926:83–91. 10.1016/j.jchromb.2013.01.037 23562906

[21] Bai F, Freeman BB III, Fraga CH, Fouladi M, Stewart CF. Determination of lapatinib (GW572016) in human plasma by liquid chromatography electrospray tandem mass spectrometry (LC–ESI-MS/MS). J Chromatogr B Analyt Technol Biomed Life Sci. 2006;831(1–2):169–75. 10.1016/j.jchromb.2005.11.044 16364699

[22] Sandra R, McM G, Martin C, Robert O. Development and application of novel analytical methods for molecularly targeted cancer therapeutics. J Chromatogr B Analyt Technol Biomed Life Sci. 2009;877:3982–90. 10.1016/j.jchromb.2009.10.008 19854117

[23] Garrido-Cano I, García-García A, Peris-Vicente J, Ochoa-Aranda E, Esteve-Romero J. A method to quantify several tyrosine kinase inhibitors in plasma by micellar liquid chromatography and validation according to the European Medicines Agency guidelines. Talanta. 2015;144:1287–95. 10.1016/j.talanta.2015.07.078 26452960

[24] Pesez M, Bartos J. Colorimetric and fluorimetrlc analysis of organic compounds. Drugs Marcel Dekker, New York 1974.

[25] Fidler AT, Baker E, Letz R. Neurobehavioural effects of occupational exposure to organic solvents among construction painters. Occup Environ Med. 1987;44(5):292–308. 10.1136/oem.44.5.292 3496112 PMC1007827

[26] Wennborg H, Bonde JP, Stenbeck M, Olsen J. Adverse reproduction outcomes among employees working in biomedical research laboratories. Scand J Work Environ Health. 2002;28(1):5–11. 10.5271/sjweh.640 11871853

[27] Lindbohm ML, Taskinen H, Sallman M, Hemminki K. Spontaneous abortions among women exposed to organic solvents. Am J Ind Med. 1990;17(4):449–63. 10.1002/ajim.4700170404 2327413

[28] Wennborg H, Bodin L, Vainio H, Axelsson G. Pregnancy outcome of personnel in Swedish biomedical research laboratories. Occup Environ Med. 2000;42(4):438–46. 10.1097/00043764-200004000-00022 10774513

[29] Kristensen P, Hilt B, Svendsen K, Grimsrud TK. Incidence of lymphohaematopoietic cancer at a university laboratory: a cluster investigation. Eur J Epidemiol. 2008;23(1):11–5. 10.1007/s10654-007-9203-5 17985198

[30] Walash M, Belal F, Tolba M, Halawa M. Micelle‐enhanced spectrofluorimetric determination of amlexanox in bioadhesive buccal tablets: application to content uniformity testing. Luminescence. 2015;30(6):823–9. 10.1002/bio.2828 25611457

[31] Ghasemi JB, Zolfonoun E. Application of principal component analysis–multivariate adaptive regression splines for the simultaneous spectrofluorimetric determination of dialkyltins in micellar media. Spectrochim Acta A Mol Biomol Spectrosc. 2013;115:357–63. 10.1016/j.saa.2013.06.054 23851178

[32] Wang CC, Masi AN, Fernández L. On-line micellar-enhanced spectrofluorimetric determination of rhodamine dye in cosmetics. Talanta. 2008;75(1):135–40. 10.1016/j.talanta.2007.10.041 18371858

[33] Berzas Nevado J, Murillo Pulgarín J, Gómez Laguna M. Spectrofluorimetric study of the β-cyclodextrin: vitamin K3 complex and determination of vitamin K3. Talanta (Oxford). 2001;53(5):951–9. 10.1016/s0039-9140(00)00580-4 18968185

[34] Transchem corporation. Kolliphor RH 40. http://transchemcorp.com/kolliphor-rh-40/, (2017) (accessed3 February 2019).

[35] Rockville M. The United States Pharmacopoeia 30, the National Formulary 25 US Pharmacopeial Convention. Electronic version. 2007.

[36] FDA, U.S. Department of Health and Human Services Food and Drug Administration Center for Drug Evaluation and Research (CDER), Guidance for industry dissolution testing of immediate release solid oral dosage forms, https://www.fda.gov/downloads/drugs/guidances/ucm070237.pdf, (1997) (accessed 3 February 2019).

[37] Leung R, Shah DO. Dynamic properties of micellar solutions: I. Effects of short-chain alcohols and polymers on micellar stability. J Colloid Interface Sci. 1986;113(2):484–99.

[38] Ferreira SC, Bruns R, Ferreira H, Matos G, David J, Brandao G, et al Box-Behnken design: an alternative for the optimization of analytical methods. Anal chim acta. 2007;597(2):179–86. 10.1016/j.aca.2007.07.011 17683728

[39] Bezerra MA, Santelli RE, Oliveira EP, Villar LS, Escaleira LA. Response surface methodology (RSM) as a tool for optimization in analytical chemistry. Talanta. 2008;76(5):965–77. 10.1016/j.talanta.2008.05.019 18761143

[40] Chemicalize, Instant cheminformatics solution, https://chemicalize.com/#/calculation=lapatinib (2019) (accessed3 February 2019).

[41] Miller J, Miller J. Calibration methods in instrumental analysis: regression and correlation. Statistics and chemometrics for analytical chemistry. 2010;6:110–53.

[42] ICH: Validation of Analytical procedures. In: Methodology (Q2AR1), International Conference on Harmonization, USA: November 1996 and November 2005. Food and Drug Administration.

